# Quality outcome of diabetes care during COVID-19 pandemic: a primary care cohort study

**DOI:** 10.1007/s00592-022-01920-0

**Published:** 2022-07-02

**Authors:** Stefania Di Gangi, Benjamin Lüthi, Laura Diaz Hernandez, Andreas Zeller, Stefan Zechmann, Roland Fischer

**Affiliations:** 1 Institute of Primary Care, University and University Hospital Zurich, Pestalozzistrasse 24, CH-8091 Zurich, Switzerland; 2 Centre for Primary Health Care, University of Basel, Basel, Switzerland; 3 Department of Endocrinology, Diabetology and Clinical Nutrition, University Hospital Zurich, Zurich, Switzerland

**Keywords:** Diabetes mellitus, Primary care, COVID-19 pandemic, Quality indicators, Diabetes outcomes

## Abstract

**Aim:**

Management of diabetes care can be affected by COVID-19 pandemic control measures. This study aimed to determine the impact of the pandemic, during 17.03.2020–16.03.2021, on quality outcomes of diabetes care in general practice in Switzerland.

**Methods:**

In this retrospective cohort study, diabetes mellitus patients (≥ 18 years) with at least one consultation at a general practitioner, during 17.03.2018–16.03.2019 (cohort 1) and 17.03.2019–16.03.2020 (cohort 2) were included and followed-up for two years. Quality indicators and outcomes of diabetes care, at patient and practitioner level, were compared before and during the pandemic. Logistic regression was performed to identify patient’s risk factors for dropout from follow-up.

**Results:**

Data from 191 practices, 23,903 patients, cohort 1 and 25,092 patients, cohort 2, were analyzed. The fraction of patients lost to follow-up, attributable to the pandemic, was 28% (95% confidence interval: 25%, 30%). During the pandemic, compared to the previous year, regular measurement of weight, HbA1c, blood pressure and serum creatinine were less frequent and less patients per practitioner reached HbA1c and blood pressure target outcomes. Factors associated with continuity of care during the pandemic were: patient age 41–80 years, longer diabetes duration, diagnosis of hypertension or dyslipidemia, influenza vaccination during the last year. Risk factors for dropout were age > 80 and receiving only insulin as anti-diabetic medication.

**Conclusion:**

A considerable quality reduction in diabetes mellitus care could be observed during the pandemic. Though the most vulnerable patients were not the most affected by the pandemic, key factors that might reduce dropout from follow-up were identified.

**Supplementary Information:**

The online version contains supplementary material available at 10.1007/s00592-022-01920-0.

## Introduction

During SARS-CoV-2 pandemic, management of chronic non-communicable diseases (NCDs) such as diabetes, hypertension, dyslipidemia can be affected in several ways. Even in the absence of an overload of COVID-19 cases, disease control measures, such as lockdown, quarantine, restrictions of public and private transport or fear of infection might have an impact on accessibility of health care [1]. In Switzerland, non-urgent patient care was suspended during lockdown (17.03.2020–26.04.2020) [2].

Several studies have examined short-term effects of COVID-19 pandemic, during or after lockdown, on glycaemic control [3–16].

Impact of COVID-19 pandemic on quality of diabetes care, as promoted through the Quality and Outcomes Framework (QOF) [17, 18], has been scarcely measured [19]. Processes and outcome indicators for type 2 diabetes patients, according to the Italian guidelines, were also compared between 2019 and 2020 [20].

This study aimed, first, to assess the impact of SARS-CoV-2 pandemic, during 17.03.2020–16.03.2021, on quality indicators and outcomes of diabetes care, based on [17, 18] and adapted for primary care in Switzerland [21–23]; second, to identify factors associated with patient dropout from follow-up during the pandemic.

## Methods

### Study design and population

This retrospective cohort study used the database from FIRE project (Family Medicine ICPC (International Classification of Primary Care) Research Using Electronic Medical Records) [24, 25] which acquires medical data from general practitioners in Switzerland.

Adult patients (≥ 18 years) with diabetes mellitus and at least one encounter at a general practitioner during baseline year: 17.03.2018–16.03.2019, cohort 1, and 17.03.2019–16.03.2020, cohort 2 (pandemic-exposed), were included and followed-up respectively before the Swiss lockdown, until 16.03.2020, and from Swiss lockdown until one year, 16.03.2021.

Diabetes mellitus diagnosis was based on one of the following conditions from whole patient history: i) at least two measurements HbA1c ≥ 6.5% (48 mmol/mol), as recommended [26]; ii) prescription of any anti-diabetic medication (Anatomical Therapeutic Chemical Classification System (ATC) [27] code A10); iii) International Classification of Primary Care 2nd edition (ICPC-2) [28] diagnosis Code T89 or T90. As Glucagon-Like-Peptide (GLP)-1 receptor agonists and Sodium-dependent Glucose Transporter 2 (SGLT-2) inhibitors could be prescribed for other reasons than diabetes (obesity and congestive heart failure or chronic kidney disease, respectively) patients treated exclusively with them were included if in addition at least once HbA1c ≥ 6.5% (48 mmol/mol).

### Data description

Patient age, in years, was defined both as continuous and categorical variable (≤ 40, 41–60, 61–80, > 80 years).

Postal code of the physician practice was used to identify urban, suburban, rural areas [29].

Comorbidities were identified through ATC codes, Global Trade Item Number (GTIN), Pharmaceutical cost groups (PCG) [30], ICPC-2 diagnosis codes [28] or laboratory measurements; details in Online Resource 1 Table 1.

Time from first diabetes diagnosis to study start was categorized as: first diagnosis during baseline, first diagnosis < 1 year, 1–5 years and > 5 years.

Insulin-dependent, non-insulin dependent and unknown was a proxy for diabetes type (Online Resource 1 Table 2).

Single diabetes medications were: Metformin, Sulfonylurea, Dipeptidylpeptidase (DPP)-4 inhibitors, SGLT-2 inhibitors, GLP-1 receptor agonists, basal insulin therapy, basal-bolus insulin therapy and other. Mixed or combination of two or more therapies were counted separately in each medication. Anti-diabetic medications were also grouped: insulin only, insulin plus oral-anti-diabetic therapy, oral-anti-diabetic (OAD) monotherapy and OAD combination therapy (Online Resource 1 Table 2).

Other medications relevant to diabetes care were: Aspirin, statins, RAAS-inhibitors. Data on influenza vaccination was also reported (Online Resource 1 Table 3).

New or not expired prescriptions, during the observation period, were included. For prescriptions without defined stop dates, a validity of 365 days was supposed, as most of diabetes patients with a particular treatment, had the same prescription in the following 12 months [31].

Indicators of diabetes care quality [21–23] were defined as proportions of patients with the following outcomes in a year interval: (1) at least two HbA1c measurements; (2) average HbA1c ≤ 7.0% (53 mmol/mol); (3) average HbA1c ≤ 8.0% (64 mmol/mol); (4) average HbA1c ≤ 9.0% (75 mmol/mol); (5) at least two blood pressure measurements; (6) average blood pressure < 140/90 mmHg; (7) at least one low density lipoprotein (LDL)-cholesterol measurement; (8) average LDL-cholesterol < 2.6 mmol/l; (9) at least one weight or body mass index (BMI) measurement; (10) at least one serum creatinine and microalbuminuria measurement.

### Statistical analysis

Baseline characteristics of each cohort: during 17.03.2018–16.03.2019 and 17.03.2019–16.03.2020 respectively, were described as number and percentage, N (%), for categorical or binary variables and as mean standard deviation (SD) for continuous variables. *χ*^2^-test, for categorical or binary variables, or *t*-test, for continuous variables, were performed for cohort comparisons or baseline and follow-up comparisons within cohorts.

Trends of all HbA1c values, weekly averaged over patients by cohort, were shown graphically.

For each cohort, from baseline to follow-up, differences between average values, for laboratory measurements, or between proportions, for quality indicators, were reported with 95% confidence interval (CI).

A subgroup analysis of outcome indicators during each year, for patients included in both cohorts, was also performed.

Quality indicator results were shown through a dumbbell plot or connected dot plot. At practice level, the median patient proportion, in each cohort, for each indicator, during baseline and follow-up periods, was reported with the interquartile range [IQR] and represented through error bar plots.

Population attributable fraction (PAF) [32] was used to compare proportions of cases in the two cohorts, with complementary outcome (dropout from follow-up, not reaching quality target …) during follow-up, considering cohort 2 being pandemic-exposed. PAF was shown graphically through a bar chart with 95% (CI) error bars.

To identify risk factors for dropout from follow-up during the first year of pandemic, for cohort 2, unadjusted and multivariable-adjusted mixed logistic regression models were performed. Random effects were considered at practice level, to correct for correlation between patients followed by the same practice. Predictors in multivariable analysis were selected with a stepwise backward approach, starting from a full model including all variables, not correlated among them, with *p* < 0.2 in univariable analysis. Results of regression analysis were reported as odds ratio (OR) (95%(CI)). Multivariable analysis results were represented through an odds ratio plot.

For all tests, *p* ≤ 0.05 was considered statistically significant. All analyses were carried out using statistical package R version 4.1.0 [33].

## Results

### Patient characteristics

**Fig. 1:**
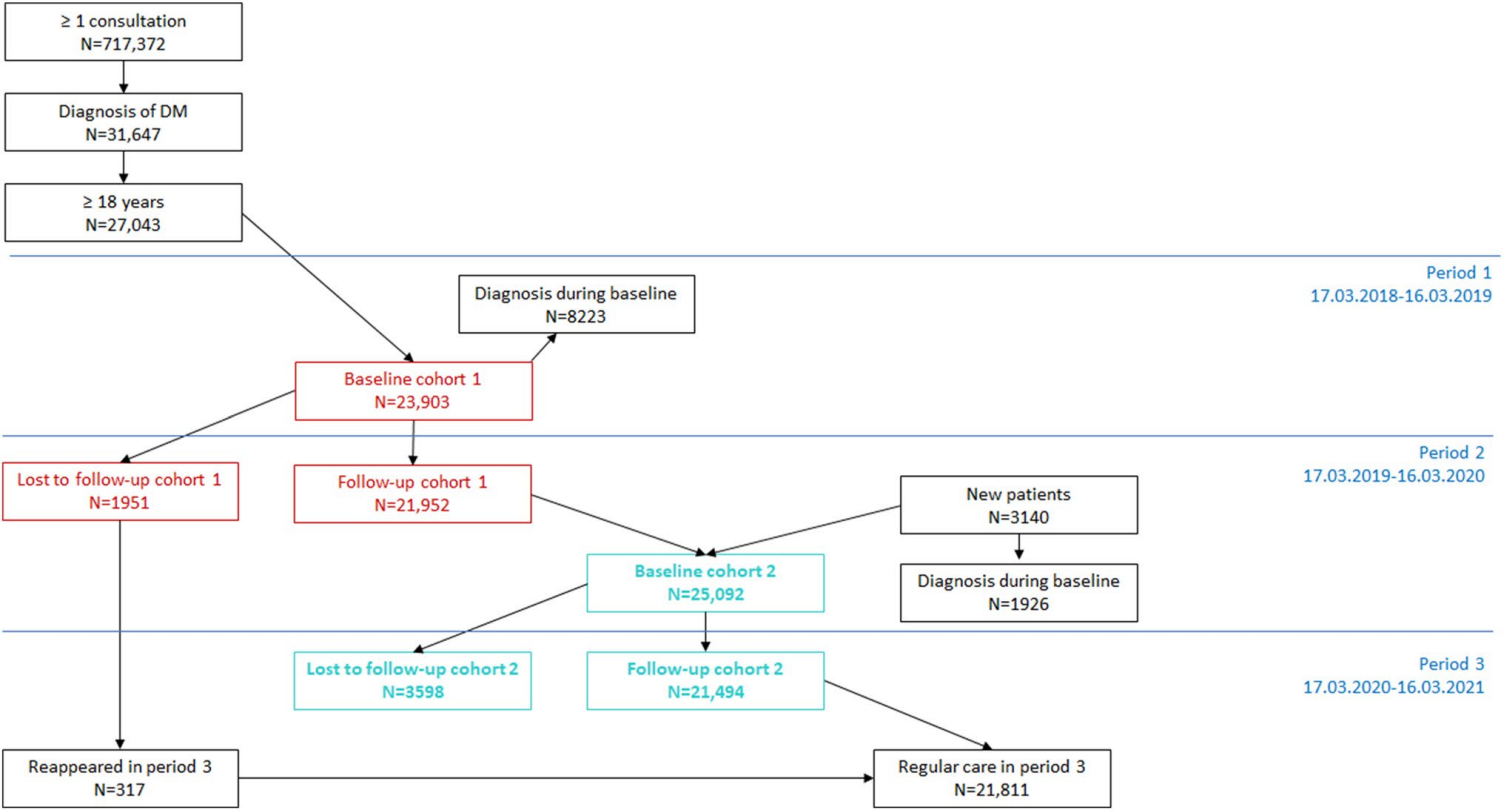
Flowchart of inclusion of 27,043 patients with diabetes mellitus. Two cohorts were identified: cohort 1, 23,903 patients, and cohort 2, 25,092 patients. Patients without an encounter in the following year were reported as lost to follow-up

A total of 27,043 patients and 191 practices were included: cohort 1, baseline 17.03.2018–16.03.2019, 23,903 patients; cohort 2, baseline 17.03.2019–16.03.2020, 25,092 patients; 21,952 patients in both cohorts, Fig. 1.

Female proportion was 43% in each cohort, *p* = 0.73. Age was 65.45(14.89) years, cohort 1, and 65.33(14.63), cohort 2, *p* = 0.38, Table 1.

**Table 1:** Patient characteristic and prevalence of quality indicators for each cohort during baseline year

	Cohort 1	Cohort 2	*p*-value
	17.03.2018–16.03.2019	17.03.2019–16.03.2020	
	*N* = 23,903	*N* = 25,092	
Age at study start: mean, SD	65.45 (14.89)	65.33 (14.63)	0.38
Female: N (%)	10,361 (43)	10,837 (43)	0.73
Area^a^ type of the GP practice: N (%)			0.23
Rural	2123 (9)	2338 (9)	
Suburban	4362 (18)	4560 (18)	
Urban	17,374 (73)	18,125 (72)	
Time from first diagnosis to study start: N(%)			< 0.001
First diagnosis during observation period	8223 (34)	9667 (39)	
< 1 years before observation period	6032 (25)	6013 (24)	
1–5 years before observation period	6907 (29)	6754 (27)	
> 5 years before the observation period	2741 (12)	2658 (11)	
Type of diabetes mellitus: N(%)			< 0.001
Insulin-dependent^b^	4274 (18)	5140 (21)	
Non-insulin dependent^c^	12,247 (51)	14,114 (56)	
Unkown^d^	7382 (31)	5838 (23)	
Diabetes medication^e^: N(%)			
Metformin (Biguanides)	11,868 (50)	14,321 (57)	< 0.001
Sulfonylurea	2440 (10)	2815 (11)	< 0.001
DPP-4 inhibitor	5230 (22)	6223 (25)	< 0.001
SGLT-2 inhibitors	2328 (10)	3543 (14)	< 0.001
GLP-1 receptor agonists	1465 (6)	2081 (8)	< 0.001
Other	282 (1)	317 (1)	0.42
Insulin^f^	4174 (17)	5051 (20)	< 0.001
Basal insulin therapy^g^	2076 (9)	2557 (10)	< 0.001
Basal-bolus insulin therapy^h^	2098 (9)	2494 (10)	< 0.001
None	8946 (37)	7248 (29)	< 0.001
Diabetes medication group: N(%)			
Insulin Only	1363 (6)	1534 (6)	0.06
Insulin + Oral anti-diabetic (OAD)	2811 (12)	3517 (14)	< 0.001
OAD monotherapy	11,760 (49)	14,334 (57)	< 0.001
OAD combinations	4359 (18)	5442 (22)	< 0.001
Other medication: N(%)			
Aspirin	6265 (26)	6977 (28)	< 0.001
Statin	9652 (40)	11,229 (45)	< 0.001
RAAS-inhibitor	12,369 (52)	14,121 (56)	< 0.001
Comorbidities: N(%)			
Hypertension	13,221 (55)	14,608 (58)	< 0.001
Dyslipidemia	9943 (42)	11,517 (46)	< 0.001
Obesity	6886 (29)	8108 (32)	< 0.001
Cardiovascular disease (CVD)	6771 (28)	7232 (29)	0.23
Chronic kidney disease	3452 (14)	4559 (18)	< 0.001
Thyroid disorders	1919 (8)	2237 (9)	< 0.001
Obstructive lung disease	2936 (12)	3252 (13)	0.02
Other	14,159 (59)	15,150 (60)	0.01
HbA1c levels ^i^: N(%)	Tot = 18,588	Tot = 19,715	
≤ 7.0% (53 mmol/mol)	10,959 (59)	11,528 (59)	0.34
≤ 8.0% (64 mmol/mol)	15,763 (85)	16,789 (85)	0.34
≤ 9.0% (75 mmol/mol)	17,504 (94)	18,623 (94)	0.22
Blood pressure < 140/90 mmHg ^i^: N (%)	9563 (54) Tot = 17,614	10,098 (56) Tot = 17,941	< 0.001
LDL-cholesterol < 2.6 mmol/l ^i^**:** N (%)	5034 (55) Tot = 9196	5635 (57) Tot = 9927	0.005
Nephropathy screening: N(%)			
≥ 1 measurement serum creatinine	15,235 (64)	15,859 (63)	0.22
≥ 1 measurement urine albumin/creatinine	2137 (9)	3220 (13)	< 0.001
Influenza vaccination: N(%)	2383 (10)	2927 (12)	< 0.001

^a^According to the Eurostat degree of urbanization classification 2011;

^b^ATC A10A or ICPC-2 T89;

^c^ATC codes other than A10A or ICPC-2 T90;

^d^ATC- or ICPC-2-codes missing;

^e^ % over all patients (not only of those with medication). Definitions provided in Online Resource 1 Table 2;

^f^ATC A10A;

^g^ATC A10AE only;

^h^ATC A10AB, A10AC and A10AD;

^i^Diabetes quality indicators

*P*-values are calculated using *χ*^2^-test for categorical variables and Student’s t-test for continuous variables. DPP-4: Dipeptidylpeptidase-4; SGLT-2: sodium dependent glucose transporter 2; GLP-1: glucagon-like peptide 1; RAAS: renin–angiotensin–aldosterone system; HbA1c: Hemoglobin A1c; LDL: low density lipoprotein

First diagnosis of diabetes mellitus occurred during baseline in 8223(34%) patients, cohort 1; 9667(39%) patients, cohort 2, *p* < 0.001.

Hypertension was the most prevalent single comorbidity: 13,221(55%) cohort 1; 14,608(58%) cohort 2, *p* < 0.001, followed by dyslipidemia, obesity, and cardiovascular diseases (such as coronary heart disease, stroke).

### Anti-diabetic and other medication prescriptions

The most prevalent therapy was OAD monotherapy: 11,760(49%) in cohort 1 and 14,334(57%) in cohort 2, *p* < 0.001 and metformin was the most used OAD: 11,868(50%), cohort 1 and 14,321(57%), cohort 2, p < 0.001.

Insulin-dependent patients were 4274(18%) in cohort 1, 5140(21%) in cohort 2.

Less patients had no anti-diabetic medication and no medication at all in cohort 2 compared to cohort 1: 7248(29%) versus 8946(37%), p < 0.001, Table 1; 3613(14%) versus 4912(20%), *p* < 0.001, Online Resource 1 Table 4.

During cohort 2 follow-up, medications prevalence was higher compared to baseline, but PAF was significant only for SGLT-2 and GLP-1 (9% and 6%), Online Resource 1 Table 5.

### Quality indicators (patient level)

**Fig. 2:**
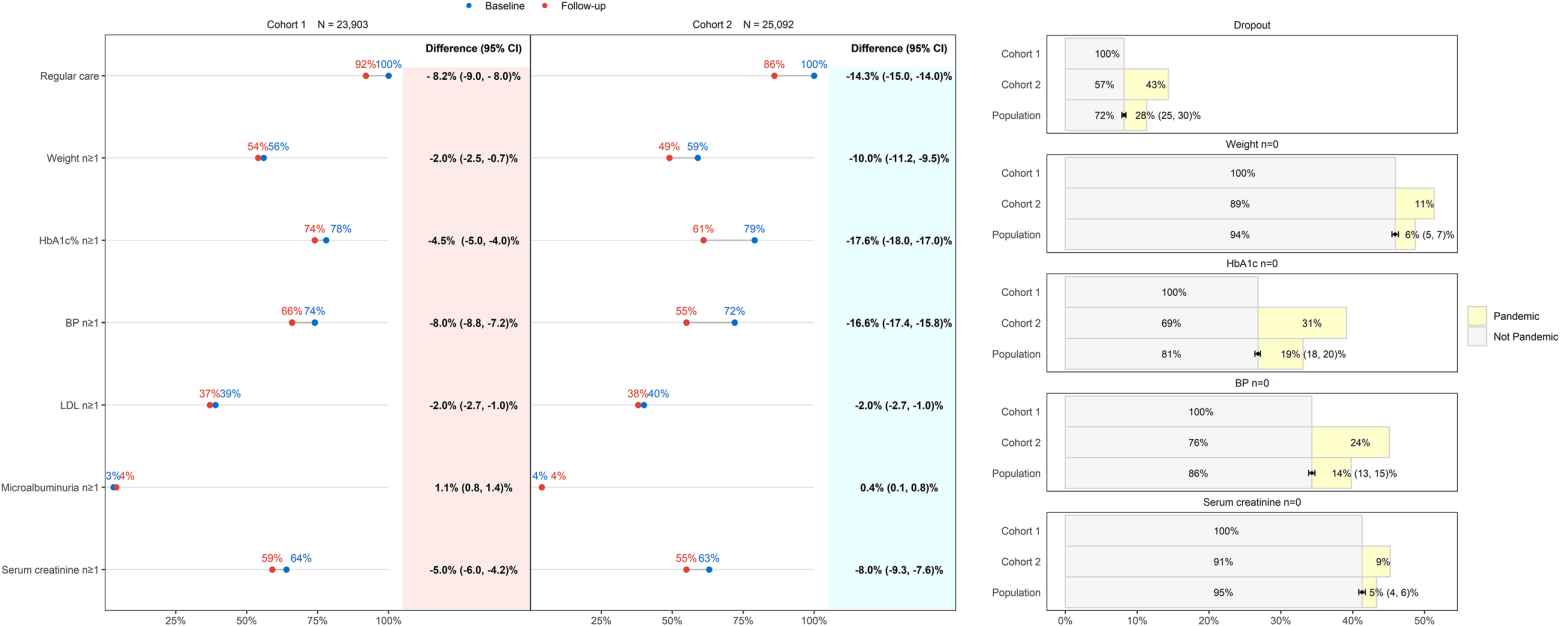
Evolution, from baseline to follow-up year, of quality indicators at patient level and impact of COVID-19 pandemic. Proportions of patients, in each year for each cohort, were represented through connected dots. Differences with 95% confidence interval (CI) were reported in columns. Attributable fraction in the exposed (cohort 2) and in the population, PAF (two cohorts), the latter with 95% confidence interval (CI), were reported in the barplot (right side). They were calculated, for the complementary outcome of each indicator in the follow-up year, as difference between the respective proportion and the proportion of unexposed cases in cohort 1. In legend, pandemic meant attributable to the pandemic exposure. Abbreviations: n: number of reported measurements per patient; HbA1c: Hemoglobin A1c; BP: blood pressure; LDL: low density lipoprotein

In cohort 1, 1951(8%) patients were lost to follow-up during 17.03.2019–16.03.2020; in cohort 2, 3598(14%) during 17.03.2020–16.03.2021; PAF 28%(25, 30)%, Fig. 2. Youngest patients, age ≤ 40 years, had the greatest PAF for dropout, 37%(30, 43)%; oldest patients, age > 80, had the lowest PAF 19%(13, 24)%, Online Resource 1 Table 5.

In cohort 1, 12,939(54%) had weight recorded during follow-up versus 13,332(56%) during baseline, difference − 2.0%(− 2.5, − 0.7)%, Fig. 2. In cohort 2, the difference was − 10%(− 11.2, − 9.5)%. PAF was 6%(5, 7)%. Average weight did not change during follow-up for each cohort, Online Resource 1 Table 6.

The proportion of patients, cohort 1, with HbA1c recorded, decreased by 4.5%(4.0, 5.0)%, absolute difference, during follow-up, starting from 78%, Fig. 2. In cohort 2, the difference was − 17.6%(− 18.0, − 17.0)% starting from 79%. PAF was 19%(18, 20)%. Average HbA1c reduced, − 0.04% (0.4 mmol/mol) difference, during follow-up in each cohort, Online Resource 1 Table 6. Weekly averages HbA1c during follow-up were higher in cohort 2, compared to cohort 1, from June 2020 to November 2020, Fig. 3.

**Fig. 3:**
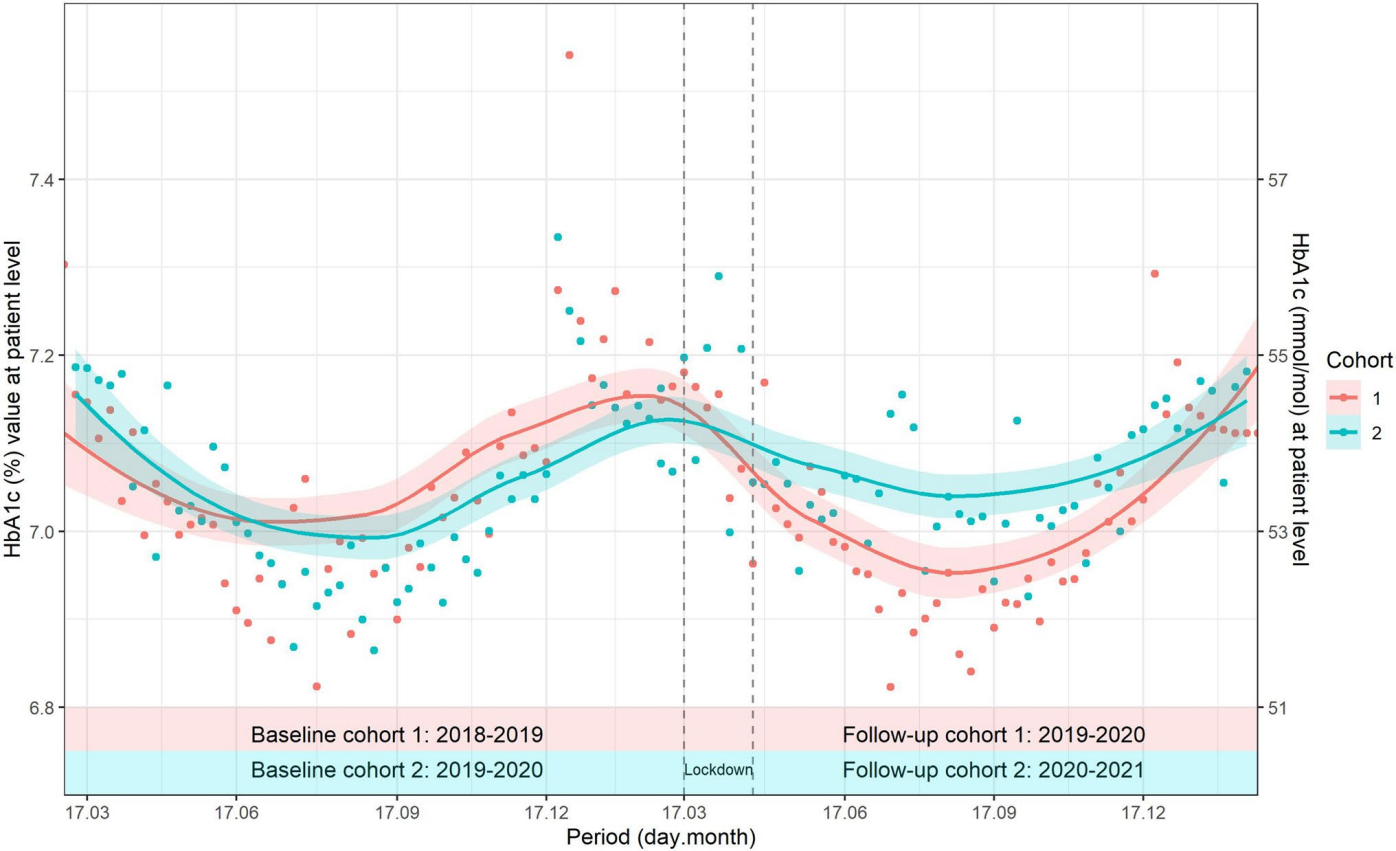
Evolution of Hemoglobin A1c (HbA1c) % values, from baseline to follow-up year for each cohort. HbA1c % were weekly averaged over patients. Points represented observed values and lines the smoothed curves. Dashed lines marked the period from 17.03.2020 to 26.04.2020, the national lockdown in Switzerland

Blood pressure was reported, during baseline, in 17,614(74%) patients in cohort 1 with a − 8.0% (− 8.8, − 7.2)% difference during follow-up; for cohort 2, the difference was − 16.6%(− 17.4, − 15.8)%, Fig. 2. PAF was 14%(13, 15)%.

As only a minority of patients had LDL-cholesterol recorded, PAF was not evaluated. However, average LDL-cholesterol improved during follow-up in each cohort, Online Resource 1 Table 6.

Patients with serum creatinine recorded during follow-up decreased more in cohort 2, than in cohort 1, Fig. 2. PAF was 5%(4, 6)%.

Since microalbuminuria was scarcely reported PAF was not evaluated.

For most indicators, around one third of patients included in both cohorts reached outcome indicator during the pre-pandemic (17.03.2018–16.03.2020) but not in the pandemic year and around one fifth of patients never reached outcome indicator during 17.03.2018–16.03.2021, Online Resource 1 Table 7.

### Quality indicators (practice level)

**Fig. 4:**
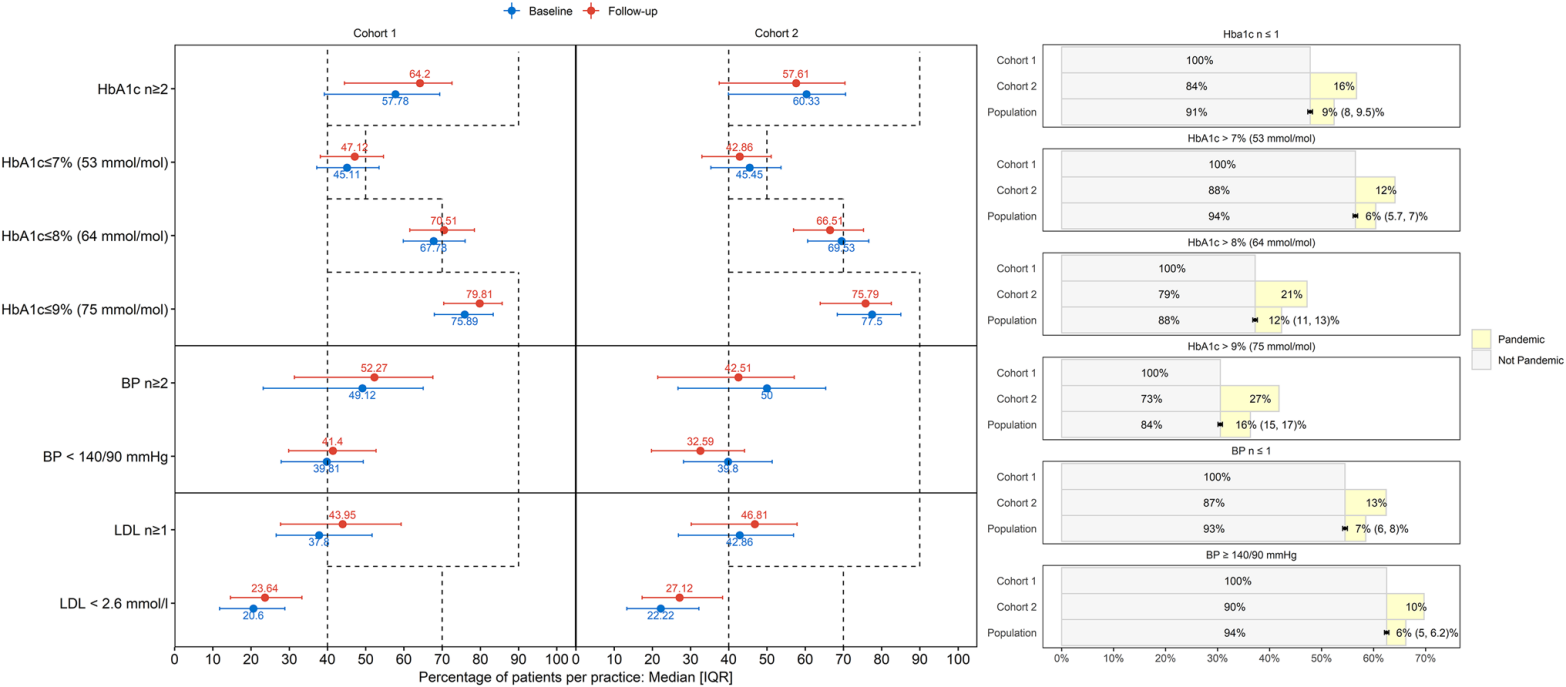
Evolution, from baseline to follow-up year, of quality indicators at practice level (Swiss Quality and Outcome Framework) and impact of COVID-19 pandemic. Median, at practice level, of the percentage of patients, for each cohort who fulfilled the indicator was reported in the error bar with the interquartile range [IQR]. Dashed line represented the quality reference area, or threshold, for each indicator. Attributable fraction in the exposed (cohort 2) and in the population, PAF (two cohorts), the latter with 95% confidence interval (CI), were reported in the barplot (right side). They were calculated, for the complementary outcome of each indicator in the follow-up year, as difference between the respective proportion and the proportion of unexposed cases in cohort 1. In legend, pandemic meant attributable to the pandemic exposure. Abbreviations: n: number of reported measurements per patient; HbA1c: Hemoglobin A1c; BP: blood pressure; LDL: low density lipoprotein

During baseline, in half the practices 76% of cohort 1 and 77% of cohort 2 patients had an average HbA1c ≤ 9.0% (75 mmol/mol); during follow-up, 80% of cohort 1 and 76% of cohort 2, Fig. 4. Of all patients with average HbA1c > 9.0% (75 mmol/mol) during follow-up, 16%(15, 17)% was the pandemic-attributable fraction. Similar numbers resulted in other HbA1c indicators but with lower PAF: 6% (5.7, 7)%, HbA1c > 7% (53 mmol/mol); 12%(11, 13)%, HbA1c > 8% (64 mmol/mol).

During baseline, in half the practices, 49% of cohort 1 and 50% of cohort 2 had blood pressure recorded at least twice; during follow-up, 52% of cohort 1 and 43% of cohort 2. PAF was 7%(6, 8)%. In half the practices, 40% of the patients, of each cohort, had an average blood pressure < 140/90 mmHg, baseline period. During follow-up, the median was 41% of cohort 1 and 33% of cohort 2. PAF was 6%(5, 6.2)%.

LDL quality indicators improved, from baseline to follow-up in each cohort, though far from the ideal threshold (dashed line). As most patients did not reach outcome targets, PAF for these outcomes were not evaluated.

### Factors associated with dropout from follow-up during the pandemic

**Fig. 5:**
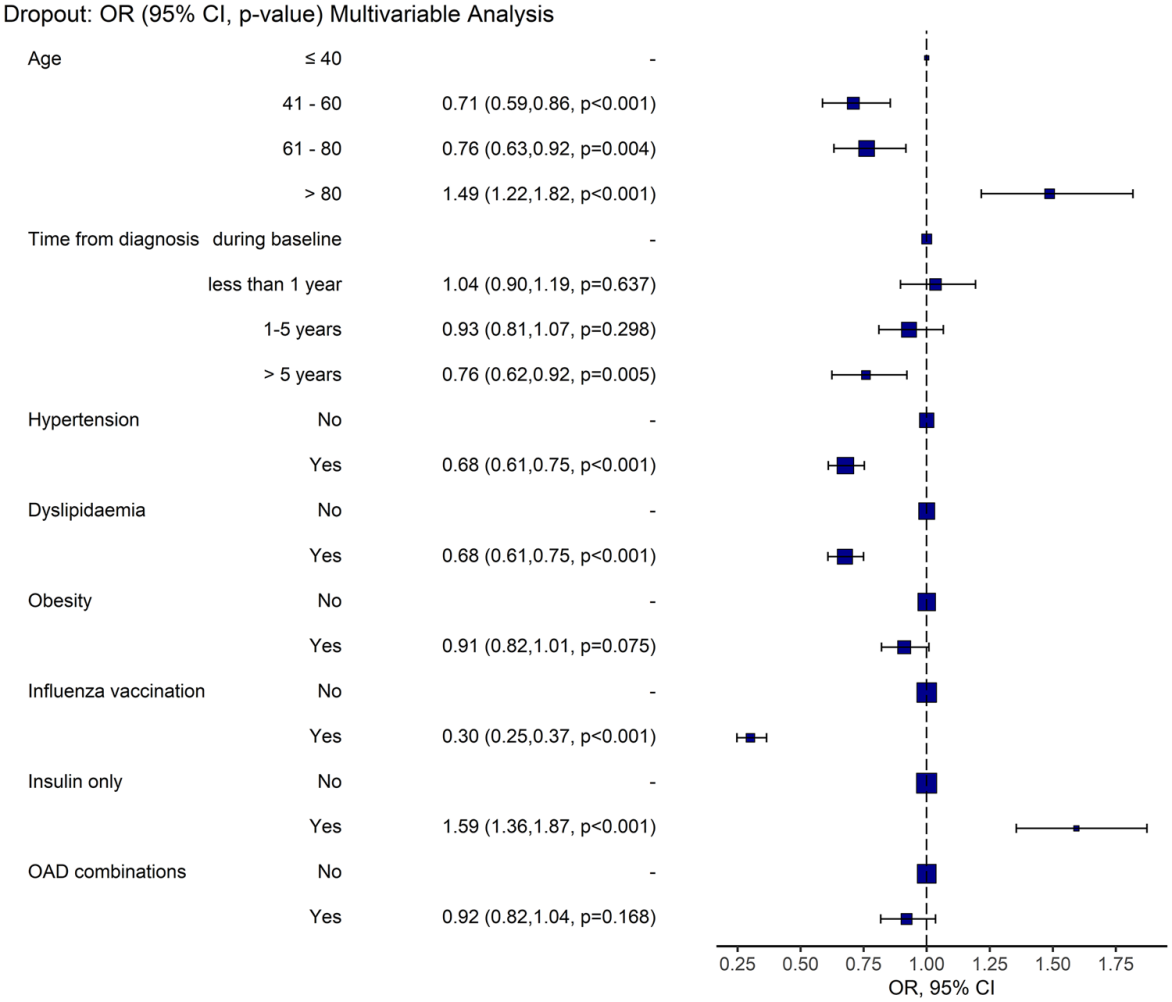
Factors associated with dropout during COVID-19 pandemic. Odds ratio (OR) plot with 95% confidence interval (CI). Multivariable mixed logistic regression analysis performed with practice as random effect. Data of cohort 2 were considered with 25,092 patients and 191 practices. Predictors were considered in only one period, 17.03.2019-16.03.2020: one value per patient

Online Resource 1 Table 8 and Fig. 5 reported results of univariable and multivariable analysis of factors associated with dropout from follow-up during the pandemic. From multivariable analysis, protective factors against dropout were: patient age 41–60, OR (95% CI), 0.71(0.59, 0.86) *p* < 0.001; age 61–80 0.76(0.63, 0.92) *p* = 0.004; time from diabetes diagnosis > 5 years 0.76(0.62, 0.92), *p* = 0.005; diagnosis of hypertension 0.68(0.61, 0.75), *p* < 0.001; diagnosis of dyslipidemia 0.68(0.61, 0.75), *p* < 0.001; influenza vaccination in previous year 0.30(0.25, 0.37), *p* < 0.001. Risk factors for dropout were: age > 80 1.49(1.22, 1.82), *p* < 0.001; receiving only insulin as anti-diabetic medication 1.59(1.36, 1.87), *p* < 0.001.

## Discussion

### Summary

In this study, the impact of COVID-19 pandemic on quality and outcomes of diabetes care was evaluated. The main findings are: (i) 28% of total dropout from follow-up during the observation period was attributable to the pandemic; (ii) the proportion of patients with regular measurement of weight, HbA1c, blood pressure and serum creatinine decreased during the pandemic compared to the previous year; (iii) at practice level, the proportion of patients reaching HbA1c and blood pressure target decreased during the pandemic compared to the previous year; (iv) factors associated with continued care during the pandemic were: patient age 41–80; longer diabetes onset; diagnosis of hypertension or dyslipidemia; influenza vaccination in the previous year. Risk factors for dropout from follow-up were age > 80 and receiving only insulin as anti-diabetic medication.

### Strengths and limitations

A strength of this study is the large database used whose validity is also supported, being the distribution of age, gender, as well as prescription proportions of anti-diabetic medications in agreement with Swiss health care settings [4, 34].

Conversely, there are some limitations. First, data quality had impact on baseline characteristics differences between the two cohorts. Moreover, we could not reliably distinguish between types 1 and type 2 patients or even be sure to have included all diabetes patients. Second, the database used (FIRE) only includes general practitioners but some of them could have a double specialty in endocrinology and general internal medicine. Moreover, some patients might have been followed by general practitioners and endocrinologists at the same time, having made their laboratory analyses at the general practitioner, but received their prescription at the endocrinologist, or vice versa. Third, we had no information about patient adherence or tolerance to treatment that could have influenced prescribing decisions. Fourth, data concerning patient-physician contact (face-to-face consultation, telephone or video-call) was unrecorded. Fifth, reason for dropout from follow-up (death, hospitalization/institutionalization, change of physician) was unknown. Sixth, prescriptions without defined stop dates might have been overestimated, supposing a validity of 365 days. Seventh, we could not examine the impact of socio-economic or life-style variables. Last, information about COVID testing or infection was missing. This could have affected dropout, increased comorbidities during the pandemic, glycaemic control or prescriptions.

### Comparison with existing literature

#### Pandemic and dropout from follow-up

During 17.03.2020–16.03.2021 the dropout rate was 14.3% of which 43% attributable to the pandemic. At population level, the pandemic-attributable fraction was 28%. Several studies found a negative impact of lockdown on consultations for diabetes patients: weekly consultations were 17.5% lower than expected without lockdown [35]; during lockdown 49% of patients did not consult their general practitioners [3], similar to [36, 37], while in India the majority reported no access to healthcare services [38]. In a large cohort of around 250′000 type 2 diabetes patients in Italy, an overall reduction of 24% in follow-up visits was observed during 2020, compared to 2019 [20]. As no study evaluated the proportion of dropout cases attributable to the pandemic, in a year time after lockdown started, our findings are not directly comparable with the existing literature.

#### Pandemic and anti-diabetic prescriptions

During the pandemic and compared to the previous year, the proportion of patients with medications increased in our study and for SGLT-2 and GLP-1 a significant effect was attributable to the pandemic. Other studies observed: an increase in insulin [39] and both insulin and OAD medications [40, 41], during the first month of pandemic as compared to the year before; a decrease in OAD during the first four months of the pandemic [42].

#### Pandemic and quality indicators of diabetes care

According to our findings, the reduction in measurement counts, for all primary care patients, was more pronounced than the reduction in consultation counts [35], though our results were at patient level and not at consultation or measurement counts. Marked reductions in the rate of health checks of type 2 diabetes patients, between March and December 2020, were highlighted [19, 20]. Accordingly, quality at patient level declined, during the pandemic, in particular for the number of patients with HbA1c recorded (17.6% absolute difference) and for the number of patients with blood pressure recorded (16.6% absolute different). However, differently from the literature, we were the first reporting the pandemic-attributable fraction for not having HbA1c and blood pressure records: PAF 19% and 14%, lesser than the one for dropout from follow-up, 28%.

Results of glycaemic decompensation, during lockdown or after few months, are conflicting [13]: no differences [3–5]; worsening [6–8, 14, 15]; improving [9–12].

We considered a larger time frame compared to these studies. Being HbA1c the most important variable of diabetes care, we analyzed weekly averaged measurements during baseline and follow-up, by cohort, evidencing around 0.1% (1.1 mmol/mol) higher values, one month after the lockdown. That means higher glucose levels during lockdown, as HbA1c correlates with mean glucose level in the previous 8–12 weeks [43], though with minor clinical impact, as after five months HbA1c returned to the previous year's level.

#### Factors associated with dropout from follow-up during pandemic

To our knowledge, this is the first study to assess association of diabetes patient characteristics and medications with dropout from follow-up during a year from the lockdown. Reduction in follow-up visits in type 2 diabetes patients was independent of age, sex, and educational level [20]. Other studies investigated the reasons to avoid or postponed the visit during lockdown [3, 40]. Differently from [20], we found that dropout of youngest patients, age ≤ 40 years, was the most affected by the pandemic, PAF 37%(30, 43)% though the oldest ones, age > 80 years, had the highest risk of dropout, after correcting for confounders. However, this higher risk was not attributable to the pandemic, since dropout of oldest patients was the less affected, PAF 19%(13, 24)%, in line with [35, 44]. Therefore, since comorbidities were associated with regular care, the most vulnerable patients remained the main focus of primary care despite the pandemic.

### Implications for research and/or practice

This study showed a decline in diabetes mellitus quality care during COVID-19 pandemic between 17.03.2020–16.03.2021, especially when facing HbA1c ≤ 7.0% (53 mmol/mol) and blood pressure < 140/90 mmHg. For most indicators, around one third of patients, included in both cohorts, reached the quality outcome during the pre-pandemic years but no during the pandemic. However, there was also a relevant proportion of patients, around one fifth for most indicators, not reaching outcome indicator in every year of observation, suggesting room for improvement in quality of diabetes care, independently of the pandemic. Though the most vulnerable patients (old, with more comorbidities) were not the most affected patients by the pandemic, our finding suggests key factors that might reduce dropout from follow-up of patients with diabetes mellitus. Primary care should have a primary role in guaranteeing continuity of care of these patients in order to prevent long-term adverse effects of the pandemic on diabetes complications.

## Supplementary Information

Below is the link to the electronic supplementary material.

Supplementary file1 (DOCX 80 KB)

## Data Availability

The datasets analyzed during the current study is available from the corresponding author on reasonable request.
