# Supplementary material for: Quality outcome of diabetes care during COVID-19 pandemic: a primary care cohort study
Source: Acta Diabetol. 2022 Jul 2;59(9):1189–200. doi: 10.1007/s00592-022-01920-0 (PMC9329414; doi:10.1007/s00592-022-01920-0)
Supplement: Supplementary file 1 — Supplementary file1 (DOCX 80 KB) [file 592_2022_1920_MOESM1_ESM.docx]

**Online Resource 1 Table 1. Comorbidity Definitions**. Each definition, at a particular time, is based on the occurrence of one of the three conditions: ICPC-2 codes, laboratory values or vital signs, medications.

| **Disease** | **Comorbidity group** | **ICPC-2 codes** | **Laboratory values or vital signs** | **Medications** |
| --- | --- | --- | --- | --- |
| Chronic Pain | Other | A01 |  | ATC= M01A, N02A, N02B |
| Secondary effect of trauma | Other | A82 |  |  |
| HIV/AIDS | Other | B90 |  | PCG = HIV/AIDS |
| Acidity related stomach problems | Other | D07  D86 D03 D85 |  | ATC = A02A, A02BA |
| Chronic constipation | Other | D12 |  | ATC = A06A and  ATC ≠ A06AX04 |
| Fecal incontinence | Other | D17 |  |  |
| Viral hepatitis | Other | D72 |  | ATC = J05AF10, J05AF11, J05AF13, J05AP, L03AB11 or  GTIN = 7680548680143,  7680548680228, 7680548690104 |
| Irritable bowel syndrome | Other | D93 |  | ATC = A06AX04,A03AA05,   or GTIN = 7680478270438, 7680478270513,  7680478270780,  7680006750012,  7680006770010,  7680301970115,  7680301970382 |
| Inflammatory bowel disease | Other | D94 |  | ATC = A07EC01, A07EC02, A07EA06 or GTIN = 7680153030265 and medication name ≠ Jorveza or Asazine |
| Liver disease | Other | D97 |  |  |
| Retinopathy | Other | F83 |  |  |
| Macular degeneration | Other | F84 |  | ATC = S01LA |
| Glaucoma | Other | F93 |  | ATC = S01E |
| Blindness | Other | F94 |  |  |
| Presbycusis/deafness | Other | H84  H86 |  |  |
| Heart disease (arrhythmias or congestive disease) | Cardiovascular disease | K77  K78 |  | ATC = C01AA05,  C01BC03, C01BC04, C01BD01, C01BD07 |
| Obstructive atherosclerotic disease (coronary, cerebral or peripheral arteries) | Cardiovascular disease | K74  K75  K76 K89  K90  K91  K92 |  | ATC = B01AC04, B01AC06, B01AC07, B01AC22, B01AC24, B01AC25, B01AC56 |
| Pulmonary heart disease | Cardiovascular disease | K82 |  | PCG= Pulmonary(arterial) hypertension |
| Hypertension | Hypertension | K85  K86  K87 | two values of BP > 140/90 mmHg | PCG= Hypertension |
| Autoimmune rheumatic diseases including rheumatoid arthritis | Other | L88 |  | PCG= Rheumatism and  ATC ≠ A07EC01 |
| Osteoarthritis | Other | L89  L90  L91 |  | ATC = M01AX25, M09AX01 |
| Osteoporosis | Other | L95 |  | ATC = M05BA, M05BB, M05BX06, A14AB01 and ATC ≠ M05BA08 |
| Poliomyelitis | Other | N70 |  |  |
| Multiple sclerosis | Other | N86 |  | PCG= Multiple sclerosis |
| Parkinson disease | Other | N87 |  | ATC = N04 |
| Epilepsy | Other | N88 |  | PCG = Epilepsy |
| Migraine | Other | N89 |  | ATC = N02C |
| Trigeminal neuralgia | Other | N92 |  |  |
| Peripheral neuritis/  neuropathy | Other | N94 |  |  |
| Alcohol abuse | Other | P15 |  | ATC = N07BB  and  ATC ≠ N07BB04 |
| Medication/drug abuse | Other | P18  P19 |  | ATC = N07BC |
| Learning disability | Other | P24 |  |  |
| Dementia | Other | P70 |  | ATC = N06D |
| Psychiatric disorders (psychotic or anxiety disorder) | Other | P72  P73  P98  P74  P75  P79  P82 |  | ATC = N05A,N05B |
| Depressive disorder | Other | P76 |  | ATC = N06A |
| Personality disorder | Other | P80 |  |  |
| Intellectual disability | Other | P85 |  |  |
| Anorexia nervosa/bulimia | Other | P86 | one value of BMI ≤ 17 and age ≤ 30 |  |
| Obstructive lung disease (asthma, COPD, or chronic bronchitis) | Obstructive lung disease | R95  R96  R79 |  | ATC = R03 |
| Psoriasis | Other | S91 |  |  |
| Chronic skin ulcer | Other | S97 |  |  |
| Obesity | Obesity | T82 | one value of BMI > 30 | ATC = A08AB |
| Thyroid diseases | Thyroid disorders | T85  T86 | two values of TSH < 0.4 or TSH > 4 | ATC = H03 |
| **Diabetes mellitus** | Diabetes mellitus | T89  T90 | two values of HbA1c ≥ 6.5%. | ATC = A10 (if only A10BJ or A10BK then included if at least one measurement of HbA1c ≥ 6.5% in the whole patient history) |
| Gout | Other | T92 |  | ATC = M04A |
| Urinary incontinence | Other | U04 |  | ATC = G04BD |
| Benign prostatic hyperplasia | Other | Y85 |  | ATC = G04C |
| Cancer | Other | A79  B72  B73  B74 B75  D74  D75  D76 D77  D78  F74  H75 K72  L71  N74  N75 N76  R84  R85  R86 R92  S77  S78  S79 T71  T72  T73  U75 U76  U77  U78  U79 W72  W73  X75  X76 X77  X78  X79  X80 X81  Y77  Y78  Y79 |  | PCG= Hormone-sensitive Cancers, Cancer, Cancer complex |
| Chronic kidney disease | Chronic kidney disease |  | GFR < 60 and (3 months before)  GFR  < 90 |  |
| Dyslipidaemia |  |  | Two of the following:  a)Triglyceride >1.7 mmol/l; b)Total Cholesterol  > 4.9 mmol/l;  c) LDL  >3 mmol/l;  d) female and HDL ≤1.2 mmol/l; f) male and HDL ≤1 mmol/l | ATC = C10 |

ICPC-2: International Classification of Primary Care, 2^nd^ edition; ATC: Anatomical Therapeutic Chemical Classification System; PCG: Pharmaceutical Cost Groups; HIV/AIDS: Human Immunodeficiency Virus/Acquired Immunodeficiency Syndrome; GTIN: Global Trade Item Number; BP: Blood Pressure; BMI: Body Mass Index; COPD: Chronic Obstructive Pulmonary Disease; eGFR: Glomerular filtration rate; TSH: Thyroid Stimulating Hormone; HbA1c: Hemoglobin A1c ; LDL: Low Density Lipoprotein; HDL: High Density Lipoprotein.

**Online Resource 1 Table 2. Diabetes medications definition.** For all medications, we considered prescriptions during an interval of 12 months.

| **Single medication** | **ATC code** |
| --- | --- |
| Metformin (Biguanides) | A10BA, A10BD01, A10BD17, A10BD13, A10BD16, A10BD15, A10BD20, A10BD23, A10BD22, A10BD18, A10BD11, A10BD26, A10BD05, A10BD14, A10BD03, A10BD10, A10BD07, A10BD02, A10BD08, A10BD25 |
| Sulfonylurea | A10BB, A10BD02, A10BD01, A10BD06, A10BD04 |
| Dipeptidylpeptidase-4 (DPP-4) inhibitors | A10BH, A10BD13, A10BD09, A10BD24, A10BD12, A10BD07, A10BD08, A10BD10, A10BD11, A10BD21, A10BD25, A10BD18, A10BD22 |
| Sodium-dependent Glucose Transporter 2 (SGLT-2) inhibitors | A10BK, A10BD15, A10BD25, A10BD21, A10BD16, A10BD19, A10BD20, A10BD23, A10BD24 |
| Glucagon-Like-Peptide 1 (GLP-1) receptor agonists | A10BJ, A10AE54, A10AE56 |
| Basal insulin therapy | A10AE and not A10AB, A10AC, A10AD |
| Basal-bolus insulin therapy | A10AB, A10AC, A10AD |
| Other | A10BF, A10BG, A10BX, A10BC, A10AF |
| **Group medication** |  |
| Insulin Only | A10A and not A10B |
| Insulin + Oral anti-diabetic (OAD) | A10A and A10B |
| OAD mono-therapy | A10BA, A10BB, A10BC, A10BH, A10BJ, A10BK, A10BX, A10BF, A10BG |
| OAD combinations | A10BD |
| **Type of diabetes patients** |  |
| Insulin-dependent | ATC A10A or ICPC-2 T89 |
| Non-insulin dependent | ATC codes other than A10A or ICPC-2 T90 |
| Unknown | ATC or ICPC-2-codes missing, only HbA1c ≥ 6.5%. |

**Online Resource 1 Table 3. Other medications and other definitions.**

| **Single/group medication** | **ATC code** |
| --- | --- |
| Aspirin | B01AC06, B01AC56 |
| Statin | C10 |
| Renin Angiotensin Aldosterone System (RAAS) inhibitor | C09 |
| **Other definitions** | **Criteria** |
| Influenza vaccination | ATC= J07BB01, J07BB02, J07BB03, J07BB04 |
| Microalbuminuria | Albumin-creatinine ratio ≥3 and ≤ 30 mg/mmol |
| estimated Glomerular Filtration Rate (eGFR) | Chronic Kidney Disease Epidemiology Collaboration (CKD-EPI) formula |

**Online Resource 1 Table 4. Additional patient characteristic during baseline period.**

|  | **Cohort 1**  **17.03.2018-16.03.2019**  **N=23,903** | **Cohort 2**  **17.03.2019-16.03.2020**  **N=25,092** | **p-value** |
| --- | --- | --- | --- |
| No. of prescribed drugs  (all drugs): N (%)  none  1  2  3 or more | 4912 (20)  4048 (17)  4276 (18)  10,667 (45) | 3613 (14)  3915 (16)  4502 (18)  13,062 (52) | <0.001 |
| No. of comorbidities: N (%)  0  1  2  3 or more | 3679 (15)  3741 (16)  3443 (14)  13,040 (55) | 2991 (12)  3843 (15)  3755 (15)  14,503 (58) | <0.001 |
| RAAS-inhibitors in patients with microalbuminuria: N (%) | Tot=595  423 (71)  no medication data 52 (9) | Tot=945  691 (73)  no medication data 55 (6) | 0.42 |
| SGLT2-inhibitors in patients with microalbuminuria: N (%) | Tot=595  107 (18) | Tot=945  203 (21) | 0.11 |
| Statin in patients > 40 years and without CVD: N (%) | Tot=15,596  4765 (31)  no medication data 3735 (24) | Tot=16,423  5842 (36)  no medication data 2672 (16) | <0.001 |
| Statin in patients with CVD:  N (%) | Tot=6771  4816 (71)  no medication data 193 (3) | Tot=7232  5310 (73)  no medication data 167 (2) | 0.003 |
| SGLT2-inhibitors and GLP-1-receptor agonists / SGLT2-inhibitors or GLP-1-receptor agonists in patients with CVD: N (%) | Tot=6771  153(2) /1280 (19) | Tot=7232  258(4) / 1744 (24) | <0.001 /  <0.001 |

p-values are calculated using χ^2^-test for categorical variables. SGLT-2: sodium dependent glucose transporter 2; RAAS: renin-angiotensin-aldosterone system; CVD: Cardiovascular diseases; GLP-1: Glucagon Like Peptide 1.

# **Online Resource 1 Table 5. Population attribution fraction (PAF) analysis for proxies of quality of care.** For each outcome**,** PAF was calculated when the proportion in the exposed population, cohort 2, was greater compared to the respective one in the not-exposed population, cohort 1. PAF was reported only when significant.

|  | **Follow-up**  **cohort 1**  **17.03.2019-16.03.2020**  **N=21,952** | **Follow-up**  **cohort 2**  **17.03.2020-16.03.2021**  **N=21,494** | **Comparison baseline/follow-up, cohort 2**  **p-value** | **PAF %**  **(95% CI)**  **Cohort 1=23,903**  **Cohort 2=25,092** |
| --- | --- | --- | --- | --- |
| Diabetes medication^a^: N(%)  Metformin (Biguanides)  Sulfonylurea  DPP-4-inhibitors  SGLT-2  GLP-1  Other  Insulin^b^  Basal insulin therapy^c^  Basal-bolus insulin therapy^d^  None | 12,326 (56)  2441 (11)  5409 (25)  3139 (14)  1862 (8)  269 (1)  4381 (20)  2220 (10)  2161 (10)   6667 (30) | 12,661 (59)  2372 (11)  5481 (26)  3944 (18)  2213 (10)   247 (1)  4552 (21)  2339 (11)  2213 (10)   5732 (27) | <0.001  0.54  0.08  <0.001  <0.001  0.28  0.005  0.02  0.21  <0.001 | 9% (7, 11)%  6% (3, 9)% |
| Diabetes medication group: N(%)  Insulin Only  Insulin + Oral anti-diabetic (OAD)  OAD mono-therapy  OAD combinations | 1256 (6)  3125 (14)  12,394 (56)  4716 (21) | 1276 (6)  3276 (15)  12,733 (59)  5105 (24) | 0.43  <0.001  <0.001  <0.001 |  |
| Other medication: N(%)  Aspirin  Statin  RAAS-inhibitors | 6259 (28)  9986 (45)  12,546 (57) | 6032 (28)  10,313 (48)  12,620 (59) | <0.001  <0.001  <0.001 |  |
| No. of all prescribed drugs: N(%)  none  1  2  3 or more | 3211 (15)  3319 (15)  3852 (17)  11,570 (53) | 2606 (12)  3178 (15)  3852 (18)  11,858 (55) | <0.001 |  |
| Comorbidities: N(%)  Hypertension  Dyslipidaemia  Obesity  Cardiovascular disease (CVD)  Chronic kidney disease  Thyroid disorders  Obstructive lung disease  Other | 13,368 (61)  10,714 (49)  7372 (34)  6727 (31)   4418 (20)  2070 (9)  3051 (14)  14,030 (64) | 13,422 (62)  11,214 (52)  7624 (35)  6622 (31)   4982 (23)  2139 (10)  2974 (14)  13,564 (63) | <0.001  <0.001  <0.001  <0.001   <0.001  <0.001  0.006  <0.001 | 4% (2, 5)% |
| No. of comorbidities: N(%)  0  1  2  3 or more | 2025 (9)  3090 (14)  3282 (15)  13,555 (62) | 1778 (8)  2874 (13)  3222 (15)  13,620 (63) | <0.001 |  |
| Influenza vaccination: N(%) | 2719 (12) | 2090 (10) | <0.001 |  |
| Dropout (subgroup analysis): N(%)  Age ≤40 years  Age 41 - 60 years  Age 61 - 80 years  Age >80 years | 205 (13)  489 (8)  800 (7)  457 (12) | 424 (29)  932 (14)  1512 (12)  730 (18) |  | 37% (30, 43)%  31% (26, 35)%  29% (25, 32)%  19% (13, 24)% |

^a^ % over all patients (not only of those with medication);

^b^ATC A10A.

^c^ATC A10AE only;

^d^ATC A10AB, A10AC and A10AD.

p-values are calculated using χ2-test for categorical variables. SGLT-2: sodium dependent glucose transporter 2; GLP-1: Glucagon Like Peptide 1; RAAS: renin-angiotensin-aldosterone system.

**Online Resource 1 Table 6. Evolution of laboratory measurements at patient level before and during the COVID-19 pandemic.**

|  | **Cohort 1**  **Baseline (n)**  **17.03.2018-16.03.2019** | **Follow-up (n)**  **17.03.2019-16.03.2020** | **Cohort 2**  **Baseline (n)**  **17.03.2019-16.03.2020** | **Follow-up (n)**  **17.03.2020-16.03.2021** |
| --- | --- | --- | --- | --- |
| Weight: mean (SD)  No. of measurements  Average value (kg) | 1.16 (1.74)  84.5 (18.49) | Change^a^ (95%-CI)  0.24 (0.21, 0.28)  -0.18 (-0.62, 0.27) | 1.50 (2.47)  84.4 (18.49) | Change (95% CI)  -0.23 (-0.27, -0.19)  -0.04 (-0.48, 0.40) |
| HbA1c: mean (SD)  No. of measurements  Average value (%)  Average value (mmol/mol) | 1.96 (2.04)  7.0 (1.21)  53.0 (13.2) | Change (95% CI)  -0.07 (-0.11, -0.03)  -0.04 (-0.07, -0.02)  -0.40 (-0.80, -0.20) | 1.98 (2.04)  7.0 (1.19)  53 (13.00) | Change (95% CI)  -0.47 (-0.51, -0.44)  -0.04 (-0.06, -0.01)  -0.4 (-0.7, -0.1) |
| Blood pressure: mean (SD)  No. of measurements  Average value  Systolic (mmHg)  Diastolic (mmHg) | 2.03 (2.35)    138 (16.83)  81 (9.96) | Change (95% CI)  0.06 (0.01, 0.10)    -0.74 (-1.09, -0.38)  -0.60 (-0.82, -0.39) | 2.21 (2.87)    137 (16.41)  80 (9.91) | Change (95% CI)  -0.56 (-0.61,-0.52)    0.55 (0.18, 0.92)  0.06 (-0.16, 0.28) |
| LDL cholesterol: mean (SD)  No. of measurements  Average value (mmol/l) | 0.50 (0.79)  2.6 (1.01) | Change (95% CI)  -0.02 (-0.03, -0.003)  -0.07 (-0.10, -0.04) | 0.52 (0.80)  2.5 (1.00) | Change (95% CI)  0.001 (-0.01, 0.02)  -0.12 (-0.15, -0.09) |
| Microalbuminuria: mean (SD)  No. of measurements  Average value (mg/l) | 0.03 (0.21)  9.4 (6.42) | Change (95% CI)  0.015 (0.01, 0.02)  -0.04 (-0.72, 0.64) | 0.05 (0.27)  9.3 (6.53) | Change (95% CI)  0.004 (-0.00, 0.01)  0.08 (-0.49, 0.65) |
| Serum creatinine: mean (SD)  No. of measurements  Average value (µmol/l)  Average value eGFR (ml/min) | 1.26 (1.61)  84 (33.69)  78 (22.90) | Change (95% CI)  -0.06 (-0.09, -0.03)   0.01 (-0.76, 0.78)  -0.34 (-0.87, 0.18) | 1.26 (1.65)  84 (33.36)  78 (23.05) | Change (95% CI)  -0.15 (-0.18, -0.12)  1.17 (0.39, 1.94)  -1.36 (-1.89, -0.84) |

HbA1c: Hemoglobin A1c; LDL: low density lipoprotein; eGFR:Glomerular filtration rate .

^a^ Change was the difference between the values in follow-up and baseline year.

**Online Resource 1 Table 7. Subgroup analysis of outcome indicators in patients of both cohorts (n=21,952).** Pre-pandemic period: 17.03.2018-16.03.2020; pandemic: 17.03.2020-16.03.2021.

| **Outcome Indicator** | N(%) of patients with outcome indicator during the pre-pandemic period but not during the pandemic | N(%) of patients with outcome indicator during each year | N(%) of patients not reaching outcome indicator in every period | N(%) patients with outcome indicator only during the pandemic |
| --- | --- | --- | --- | --- |
| **HbA1c** | | | | |
| at least two HbA1c measurements | 7802 (36) | 8560 (39) | 4629 (21) | 961 (4) |
| average HbA1c≤7.0%  (53 mmol/mol) | 6928 (32) | 6728 (31) | 7112 (32) | 1184 (5) |
| average HbA1c≤8.0%  (64 mmol/mol) | 7282 (33) | 10,749 (49) | 3071 (14) | 850 (4) |
| average HbA1c≤9.0%  (75 mmol/mol) | 7208 (33) | 12,161 (55) | 1974 (9) | 609 (3) |
| **Blood pressure** | | | | |
| at least two blood pressure measurements | 7180 (33) | 7218 (33) | 6485 (29) | 1069 (5) |
| average blood pressure<140/90 mmHg | 4700 (22) | 7753 (35) | 8173 (37) | 1326 (6) |
| **Low density lipoprotein (LDL)-cholesterol** | | | | |
| at least one LDL measurement | 6213 (28) | 6334 (29) | 7492 (34) | 1913 (9) |
| average LDL-cholesterol<2.6 mmol/l | 2151 (10) | 5307 (24) | 12,759 (58) | 1735 (8) |
| **Weight or body mass index (BMI)** | | | | |
| at least one weight BMI measurement | 7068 (32) | 9485 (43) | 4186 (19) | 1213 (6) |
| **Serum creatinine** | | | | |
| at least one serum creatinine | 7046 (32) | 10,920 (50) | 2824 (13) | 1162 (5) |

**Online Resource 1 Table 8. Patient level factors associated with dropout during the first year of COVID-19 pandemic**. Data of cohort 2 are considered. Logistic univariable mixed model with practice as random effect was performed.

| **Independent variable (reference category)** | **Univariate Analysis**  **OR (95% CI)** | **p-value** | **N Patients** | **N Practices** |
| --- | --- | --- | --- | --- |
| Age (≤40 years)  41- 60  61- 80  >80 | 0.49 (0.41,0.59)  0.43 (0.36,0.52)  0.83 (0.69,1.01) | <0.001  <0.001  0.06 | 25,092 | 191 |
| Gender male (female) | 0.99 (0.91,1.08) | 0.88 | 25,088 | 191 |
| Area type of the GP practice^a^ (urban)  suburban  rural | 0.83 (0.53, 1.3)  0.74 (0.41,1.32) | 0.41  0.31 | 25,023 | 190 |
| Type of diabetes  (insulin-dependent)  non-insulin dependent  Not known | 0.78 (0.7, 0.87)  0.96 (0.83, 1.12) | <0.001  0.63 | 25,092 | 191 |
| Time from first DM diagnosis (during observation period)  less than 1 year  1-5 years  >5 years | 0.93 (0.81,1.07)  0.76 (0.66,0.86)  0.57 (0.47,0.69) | 0.30  <0.001  <0.001 | 25,092 | 191 |
| No anti-diabetic medication (any) | 1.11 (0.99,1.25) | 0.06 | 25,092 | 191 |
| No medication (any) | 1.56 (1.36,1.78) | <0.001 | 25,092 | 191 |
| Diabetes single medication^b^  Metformin  Sulfonylurea  DPP-4 inhibitors  SGLT-2 inhibitors  GLP-1 receptor agonists  Basal insulin therapy  Basal-bolus insulin therapy | 0.71 (0.64,0.78)  1.00 (0.87,1.15)  0.87 (0.79,0.97)  0.66 (0.58,0.76)  0.83 (0.71,0.98)  1.01 (0.88,1.16)  1.40 (1.22, 1.60) | <0.001  0.96  0.01  <0.001  0.03  0.89  <0.001 | 25,092 | 191 |
| Diabetes medication group^b^  Insulin Only  Insulin + OAD  OAD mono-therapy  OAD combinations | 1.80 (1.55,2.11)  0.94 (0.83,1.07)  0.77 (0.70,0.85)  0.76 (0.68,0.85) | <0.001  0.35  <0.001  <0.001 | 25,092 | 191 |
| HbA1c levels (<7% (53 mmol/mol))  ≥7.0 and <8.0% (≥53 and <64 mmol/mol)  ≥ 8.0 and ≤9.0% (≥64 and ≤75 mmol/mol)  >9.0% (75 mmol/mol) | 1.07 (0.93,1.23)  1.29 (1.06,1.57)  1.61 (1.27,2.03) | 0.34  0.01  <0.001 | 19,715 | 188 |
| Other medications^b^  Aspirin  Statin  RASS | 0.81(0.73, 0.90)  0.75 (0.68,0.82)  0.70 (0.64,0.77) | <0.001  <0.001  <0.001 | 25,092 | 191 |
| Comorbidities^b^  Hypertension  Dyslipidaemia  Obesity  Cardiovascular disease  Chronic kidney disease  Thyroid disorders  Obstructive lung disease | 0.56 (0.51,0.62)  0.53 (0.48,0.58)  0.75 (0.68,0.83)  0.77 (0.69,0.85)  0.95 (0.84,1.06)  0.93 (0.79,1.09)  0.82 (0.71,0.94) | <0.001  <0.001  <0.001  <0.001  0.35  0.37  0.006 | 25,092 | 191 |
| No. of comorbidities (none)  1  2  3 or more | 0.64 (0.55,0.74)  0.40 (0.34,0.46)  0.32 (0.28,0.36) | <0.001  <0.001  <0.001 | 25,092 | 191 |
| Average blood pressure <140/90mmHg in the baseline year (average ≥140/90 mmHg) | 1.02 (0.90,1.15) | 0.73 | 17,941 | 187 |
| Average LDL-cholesterol < 2.6 mmol/l in the baseline year (average ≥2.6 mmol/l) | 0.62 (0.50,0.76) | <0.001 | 9927 | 184 |
| Influenza vaccination within the last year (no) | 0.30 (0.25,0.37) | <0.001 | 25,092 | 191 |

^a^ According to the Eurostat degree of urbanization classification 2011.

^b^ For any category of this predictor, the reference was the complementary group.

DPP-4: Dipeptidylpeptidase-4; SGLT-2: sodium dependent glucose transporter 2; GLP-1: glucagon-like peptide 1; HbA1c: Hemoglobin A1c; RAAS: renin-angiotensin-aldosterone system; LDL: low density lipoprotein.
